# Omics Technologies and Neovascular Ocular Disorders

**DOI:** 10.1155/2014/231348

**Published:** 2014-05-26

**Authors:** Daniel Petrovič, Quan Dong Nguyen, Borut Peterlin, Goran Petrovski

**Affiliations:** ^1^Faculty of Medicine, University of Ljubljana, 1000 Ljubljana, Slovenia; ^2^Stanley M. Truhlsen Eye Institute, University of Nebraska Medical Center, Omaha, NE 68198, USA; ^3^Institute of Medical Genetics, Department of Obstetrics and Gynecology, University Medical Centre, Zaloška 2, 1000 Ljubljana, Slovenia; ^4^Department of Ophthalmology, University of Szeged, Szeged 6700, Hungary

Neovascular ocular disorders such as diabetic retinopathy and neovascular age-related macular degeneration (neovascular AMD) are an important cause of blindness in the world [1]. In these disorders, neoangiogenesis plays an important role [2], while various environmental and genetics factors are involved in their pathogenesis [2]. In the last decade of research, the importance of gene-environmental interactions and epigenetic mechanisms has been increasingly emphasized.

Inflammation has an important role in the development of proliferative diabetic retinopathy (PDR) as described in the paper by M. Urbančič et al., 2013, “*A flow cytometric analysis of vitreous inflammatory cells in patients with proliferative diabetic retinopathy.*” Histological and flow cytometric analyses have recently demonstrated the importance of histiocytes/macrophages and T lymphocytes in the development and activation of this disease, but no prediction on the visual prognosis was made. Moreover, higher CD4/CD8 ratio in the vitreous of patients with PDR compared to that in their blood was consistent with local inflammatory response in the disease.

Characterization of the cell surface marker phenotype of* ex vivo *cultured cells growing out of human fibrovascular epiretinal membranes (fvERMs) from PDR can give insight into their function in immunity, angiogenesis, and retinal detachment, as described in the paper by Z. Veréb et al., 2013,* “Functional and molecular characterization of ex vivo cultured epiretinal membrane cells from human proliferative diabetic retinopathy.*” Several surface markers such as hematopoietic (CD34, CD47) and mesenchymal stem cell markers (CD73, CD90/Thy-1, and PDGFR *β*) have recently been reported in fvERMs from patients with PDR. Additionally, secretion of different angiogenesis-related factors (DPPIV/CD26, EG-VEGF/PK1, ET-1, IGFBP-2 and 3, IL-8/CXCL8, MCP-1/CCL2, MMP-9, PTX3/TSG-14, serpin E1/PAI-1, serpin F1/PEDF, TIMP-1, and TSP-1) was demonstrated in cells growing out of the fvERMs.

The importance of different genes in the pathogenesis of PDR has been reviewed by D. Petrovič, 2013, in the paper* “Candidate genes for proliferative diabetic retinopathy.*” Several pathogenetic mechanisms have been implicated in the development of PDR such as alteration in retinal blood flow, hemostatic abnormalities, metabolic changes, increased oxidative stress, increased polyol and hexosamine pathway flux, activation of protein kinase C isoforms, and increased advanced glycation end-product formation, growth factors, and so forth (D. Petrovič, 2013,* “Candidate genes for proliferative diabetic retinopathy”* [1]). One of the regulatory genes that has been implicated in the development of diabetic retinopathy is osteoprotegerin acting as an important regulatory molecule in the vasculature, as shown in the paper by S. M. Ramuš et al., 2013, “*SNP rs2073618 of the osteoprotegerin gene is associated with diabetic retinopathy in Slovenian patients with type 2 diabetes.”*

Epigenetic mechanisms are expected to be involved in the pathogenesis of PDR as well. Gene polymorphisms and epigenetic mechanisms responsible for PDR are reviewed in this paper (D. Petrovič, 2013, “*Candidate genes for proliferative diabetic retinopathy*”; R. A. Kowluru et al., 2013, “*Epigenetic modifications and diabetic retinopathy*”). The role of epigenetics in diabetic retinopathy is now an emerging area as described in “*Epigenetic modifications and diabetic retinopathy*” by R. A. Kowluru et al., 2013. It is well known that diabetic environment facilitates epigenetics modifications, which can alter the gene expression without permanent changes in DNA sequence. It has been shown recently that genes encoding mitochondrial superoxide dismutase and matrix metalloproteinase-9 are epigenetically modified, and, by activation of epigenetically modified enzymes, DNA methyltransferases are increased and micro-RNAs responsible for regulating nuclear transcriptional factor and growth factors are upregulated.

Additionally, omics analysis provides important information that enables the development of new treatment modalities in vascular eye disorders as described in the paper by A. Maver et al., 2013, “*Integration of data from omic studies with the literature-based discovery towards identification of novel treatments for neovascularization in diabetic retinopathy.*” Discovery of novel treatments for diabetic retinopathy by complementing the interpretation of omics results using the vast body of information existing in the published literature is a promising approach. With collection of data from transcriptomic studies performed on retinal tissue from animal models of diabetic retinopathy, identification of altered genes and pathways can be enabled, and this approach may help determine new therapies in diabetic retinopathy.

In the development of AMD, one of the leading causes of blindness in the elderly, many environmental, lifestyle, and genetic factors are involved. Among them, oxidative stress seems to play a pivotal role. The response to oxidative stress involves several cellular defense reactions leading to the accumulation of detrimental products such as intracellular lipofuscin and extracellular drusen. Moreover, there are many anatomical changes in the retinal pigment epithelium, Bruch's membrane, and choriocapillaris in response to chronic oxidative stress, hypoxia, and disturbed autophagy and these are estimated to be crucial components in the pathology of neovascular processes in AMD, as shown in the paper by J. Blasiak et al., 2013, “*Oxidative stress, hypoxia, and autophagy in the neovascular processes of age-related macular degeneration*.”

The disease has remained at the epicenter of clinical research in ophthalmology. During the past decade, the focus of researchers has ranged from understanding the role of vascular endothelial growth factor (VEGF) in the angiogenic cascades to developing new therapies for retinal vascular diseases. Anti-VEGF agents such as ranibizumab and aflibercept have become increasingly well-established therapies as shown in the paper by M. Hanout et al., 2013,* “Therapies for neovascular age-related macular degeneration: current approaches and pharmacologic agents in development.*” Additionally, many other new therapeutic agents, which are in the early phase of clinical trials, have shown promising results.

Stem cell therapy is a promising approach in different diseases, including those causing blindness. Recent reports of retinal stem cells being present in several locations of the adult eye have sparked great hopes that they may be used to treat the millions of people worldwide who suffer from blindness as a result of retinal disease or injury [3]. A population of proliferative cells derived from the ciliary body epithelium has been considered one of the prime stem cell candidates, and as such they have received much attention in recent years ([3] “R. Frøen et al., 2013, “*Does the adult human ciliary body epithelium contain “true” retinal stem cells?*”). However, until fully accepted as an important treatment modality in different eye disorders, their usefulness must be confirmed in well-designed clinical trials.

*Daniel Petrovič* *Quan Dong Nguyen* *Borut Peterlin* *Goran Petrovski*
